# Assessment of the Correlation Between Systemic Conditions and Pulp Canal Calcification: A Case-Control Study

**DOI:** 10.7759/cureus.45484

**Published:** 2023-09-18

**Authors:** Ruaa A Alamoudi, Fatimah M Alzayer, Rawabi A Alotaibi, Faisal Alghamdi, Shatha Zahran

**Affiliations:** 1 Endodontics, Faculty of Dentistry, King Abdulaziz University, Jeddah, SAU; 2 General Dentistry, Faculty of Dentistry, King Abdulaziz University, Jeddah, SAU; 3 Oral Biology, Faculty of Dentistry, King Abdulaziz University, Jeddah, SAU

**Keywords:** systemic medications, systemic-diseases, smoking habits, dental pulp stone, pulp canal obliteration, pulp canal calcification

## Abstract

Objective: Pulp canal calcification is a dental condition that is characterized by the deposition of mineralized tissue within the dental pulp space. While it is primarily a local phenomenon, recent studies have suggested a potential link between pulp calcification and systemic diseases. This study aimed to determine the correlation between certain systemic diseases, medications, and the presence of pulp canal calcification. Second, it aimed to estimate the prevalence of pulp calcification in the smoker population.

Methods: A pair-matched case-control observational study was conducted from June 2022 to June 2023 at the Faculty of Dentistry, King Abdulaziz University. Digital periapical and bitewing radiographs were used for case-based sampling. Patients were categorized into two study groups: the cases group (n=100), consisting of patients with pulp canal calcification including either pulp stone, pulp canal obliteration, or both. Whereas the control group (n=100), consisted of patients without pulp canal calcification. Detailed medical histories were obtained to identify the presence of systemic diseases. Additionally, systemic medications and smoking status were documented.

Result: The participants were divided into two groups; cases (n=100), diagnosed with teeth calcifications and confirmed by radiographic examination, and controls (n=100), showing no evidence of teeth calcifications. Among the patients in the calcification group, 26% had a history of systemic disease compared to 17% in the control non-calcification group with no significant difference between both groups. No correlation between certain medications and pulp canal calcification. Smoking did not demonstrate a statistically significant association with teeth calcifications (p > 0.05).

Conclusion: Pulp calcification on routine radiographic examination could indicate an underlying unnoticed systemic disorder that demands accurate referral and subsequent therapy.

## Introduction

Pulp canal calcification is a dental condition that is characterized by the deposition of mineralized tissue within the dental pulp space which leads to narrowing or even obliteration of the pulp tissue [1]. According to Kronfeld, there are two forms of calcification that occur in the pulp space; calcification in the radicular pulp is referred to as pulp canal obliteration, whereas calcification in the coronal region is referred to as pulp stone [2]. The mechanism of canal calcification is not fully understood, yet it is generally accepted to be related to damage to the neurovascular supply of the pulp [3]. Pulp calcification occurs as a result of various factors such as aging, caries, dental trauma, and orthodontic treatment [4,5].

While it is primarily a local phenomenon, recent studies have suggested a potential link between pulp calcification and systemic diseases. It is hypothesized that pulp canal calcification may be a marker of systemic calcification which is a common feature of many systemic diseases. One study suggested that pulp canal calcification may be associated with cardiovascular conditions such as coronary artery diseases and atherosclerosis [6]. Another study found that patients with pulp canal calcification had a higher prevalence of hypertension and hyperlipidemia compared to those without pulp calcification [7]. Moreover, an association between pulp canal calcification and renal stones was reported [8]. Furthermore, several studies concluded a strong association between canal calcification and diabetes mellitus [9,10].

Medications can contribute to the development and progression of pulp canal calcification. One study reported that systemic administration of statins is a risk factor for the development of pulp canal calcification [11]. In addition, long-term usage of glucocorticoids contributed significantly to the onset of pulp canal calcification [12].

Smoking is a major risk factor for various dental and oral health problems [13]. Smoking can cause chronic inflammation in the oral cavity, which can contribute to the development and progression of pulp canal calcification [14]. The harmful chemicals in cigarette smoke can damage the blood vessels in the oral cavity, leading to reduced blood flow to the teeth and gums [15]. This reduction in blood flow can result in a decrease in oxygen and nutrient supply to the teeth, which can cause inflammation and damage to the dental pulp. Chronic inflammation can lead to the deposition of calcium salts in the pulp chamber and root canal space, resulting in pulp canal calcification [16]. A study has shown a strong association between smoking and pulp canal calcification. Prasada and Issac reported a positive correlation between tobacco chewers and pulp stones [17].

To date, there is no strong evidence to support the correlation between certain systemic diseases, medications, smoking, and pulp canal calcification. This study primarily aimed to determine if there is any correlation between systemic diseases, medications, and the presence of pulp canal calcification. Second, it aimed to estimate the prevalence of pulp calcification in the smoker population. The null hypothesis advocated there is a strong correlation between systemic factors such as medical diseases, medications, smoking, and pulp calcification while the alternative hypothesis reported no correlation between systemic factors and pulp calcification. 

## Materials and methods

Study description

This pair-matched case-control observational study was conducted at the Faculty of Dentistry, King Abdulaziz University, Jeddah, Saudi Arabia from June 2022 to June 2023. Ethical approval from the institutional ethics committee of King Abdulaziz University was obtained prior to the initiation of the study (reference number 331-11-21). Informed written consent was obtained from all patients before patients’ examination.

Sample size calculation

Sample size calculations were performed using STATA v15 software (StataCorp LLC, College Station, TX, USA). In a matched case-control study with a total sample size of 200 patients (100 cases and 100 controls), a two-sided 5% significance test for the null hypothesis that the odds ratio of the association between pulp calcification and systemic diseases was equal one and have 80.19% power to find an odds ratio of 2 (i.e., systemic diseases doubles the risk of pulp calcification development) when the control exposure proportion is 0.2 (the assumption here is that 20% of the subjects will have systemic disease) the correlation between cases and controls based on the matching criteria is 0.2.

Data collection

Data collection was carried out from August 2022 to March 2023. Patients seeking multiple restorative dental treatments at the dental clinics in King Abdulaziz University were included in the study. Digital periapical and bitewing radiographs were used for case-based sampling. Patients were categorized into two study groups; the cases group, consisting of patients with pulp canal calcification including either pulp stone, pulp canal obliteration or both, and the control group, subjects with no radiographic signs of pulp stones or canal calcification. Patients under 18 years of age, inadequate quality radiographs, and root canal-treated teeth were excluded from the study. Demographic variables such as age and gender were recorded, and detailed medical histories were obtained to identify the presence of systemic diseases, including cardiovascular diseases, diabetes mellitus, renal diseases, and autoimmune disorders. Additionally, systemic medications and smoking status were documented.

Radiographic examination

**Figure 1:**
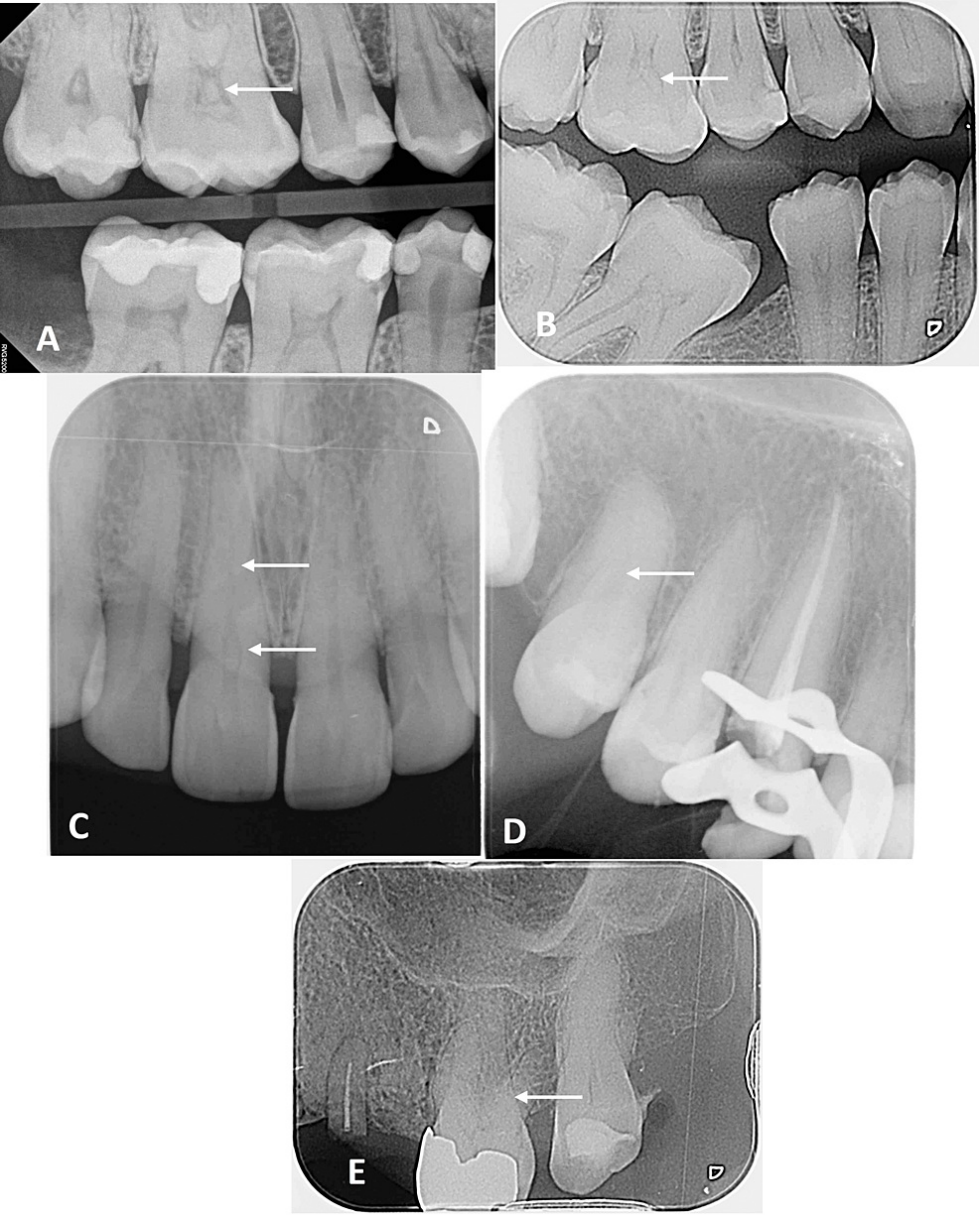
Radiographic images showing different types of pulp stone and pulp canal obliteration A: Bitewing radiograph of tooth 16 revealed pulp stone that partially occlude the pulp chamber; B: Bitewing radiograph of tooth 16 revealed pulp stone that completely occlude the pulp chamber; C: Periapical radiograph of tooth 11 revealed both pulp stone that partially occlude the pulp chamber and partial pulp canal obliteration; D: Periapical radiograph of tooth 15 revealed complete obliteration of the pulp chamber and pulp canal obliteration where the canal space is almost invisible; E: Periapical radiograph of tooth 26 revealed diffused radio-opacities throughout the pulp chamber and the canal outlines for all roots were quite unclear and cannot be traceable till the apex.

Periapical and bitewing radiographs were taken with size #1 or #2 digital plates using FOCUSTM Intraoral X-ray unit (KaVo Dental, Charlotte, NC, USA). Exposure parameters were set at kVp = 70 and mA = 7, with an average exposure time of 1 second. The Gendex DenOptix Digital Imaging System (Gendex, York, PA, USA) was utilized for image viewing in a dimmed room. Two experienced dentists assessed the radiographic images. A tooth was classified as having a pulp stone if one or multiple radiopaque masses was in the pulp chamber, while pulp canal obliteration was determined based on narrowing or complete closure of the root canal space seen on conventional radiograph (Figure 1).

The examiners underwent calibration, which involved evaluating 50 images of calcified teeth not included in the current study. To minimize examiner fatigue, viewing sessions were divided into multiple periods within the day, with random ordering of images. Intra-examiner reliability was assessed by re-evaluating the same 50 images in a separate session conducted two weeks apart.

Statistical analysis

Data were analyzed using the Statistical Package for Social Sciences (SPSS), version 22.0 (IBM Corp., Armonk, NY, USA). The significance level was set at 5% (α=0.05). The two groups were matched by age and gender. Inter- and intra-examiner reliability were assessed using Cohen's kappa correlation coefficient. Descriptive statistics were used to summarize the study characteristics. Pearson's chi-square test was employed to determine the association between systemic factors such as diseases, medications, smoking, and the presence of pulp calcification. Crude odds ratios (ORs) and their respective 95% confidence intervals (CIs) were calculated to evaluate the association between calcification and multiple factors.

## Results

**Figure 2:**
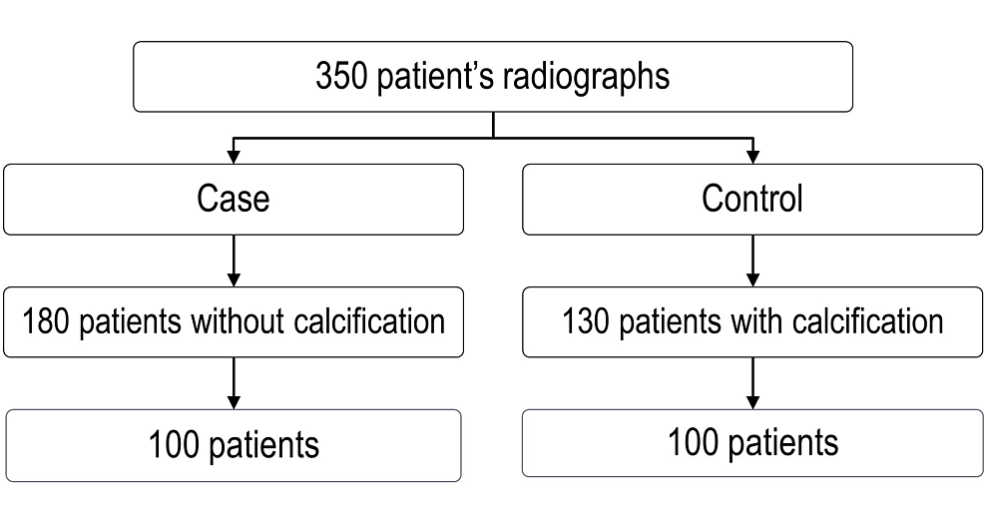
Flow chart of patients’ recruitment for a case-control study

A total of 350 patient’s radiographs were initially reviewed. Among them, 180 patients without calcification and 130 patients with calcification met the inclusion criteria and consented to participate in the study. Following age and gender matching, the study comprised 200 participants with an age range of 18 to 60 years old. The participants were divided into two groups: cases (n=100), diagnosed with teeth calcifications and confirmed by radiographic examination, and controls (n=100), showing no evidence of teeth calcifications (Figure 2).

Inter- and intra-examiner reliability measures demonstrated strong agreement, with kappa scores of 0.88 and 0.89 respectively. The average age of the patients was 32.3 years, and the sample consisted of 76 males and 124 females. The distribution of systemic variables in relation to calcification is presented in Table 1.

**Table 1:** Overall prevalence and comparative analysis of case and control groups among different systemic variables

	Patients without calcifications n (%)	Patients with calcifications n (%)	p values
Age group			
Less than 20 years old	16 (16.0%)	16 (16.0%)	1.000
21-30	41 (41.0%)	41 (41.0%)	
31-40	15 (15.0%)	15 (15.0%)	
Above 40	28 (28.0%)	28 (28.0%)	
Gender			
Male	38 (38.0%)	38 (38.0%)	1.000
Female	62 (62.0%)	62 (62.0%)	
Medical history			
Healthy	83 (83.0%)	74 (74.0%)	0.281
Anemia	2 (2.0%)	3 (3.0%)	
Asthma	2 (2.0%)	3 (3.0%)	
Colon diseases	1 (1.0%)	0	
Diabetes	6 (6.0%)	10 (10.0%)	
Hyperlipidemia	1 (1.0%)	0	
Hypertension	0	4 (4.0%)	
Irritable bowel diseases	0	1 (1.0%)	
Mental diseases	0	1 (1.0%)	
Seizure	2 (2.0%)	0	
Thyroid diseases	3 (3.0%)	4 (4.0%)	
Medical history			
Yes	17 (17.0%)	26 (26.0%)	0.121
No	83 (83.0%)	74 (74.0%)	
Medications			
Yes	31 (31%)	28 (28%)	
No	69 (69%)	71 (71%)	0.125
Smoking			
Yes	27 (27.0%)	24 (24.0%)	0.626
No	73 (73.0%)	76 (76.0%)	
Total	100	100	

**Figure 3:**
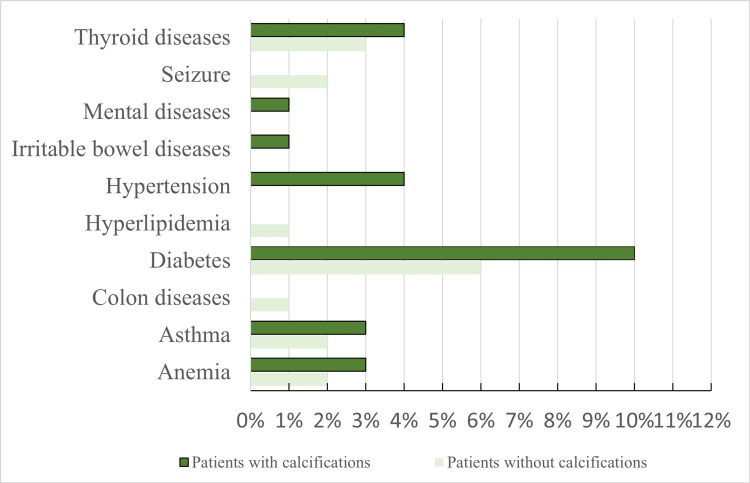
Distribution of systemic diseases among patients with calcifications and without calcifications (n=100) in each group

Among the patients in the calcification group, 26 (26%) had systemic conditions, including hypertension, diabetes mellitus, hyperlipidemia, anemia, asthma, colon diseases, irritable bowel diseases, mental diseases, seizure, and thyroid diseases, compared to 17 (17%) in the control non-calcification group with no significant difference between both groups. Diabetes mellitus was observed in 10 (10%) of the case group and 6 (6%) of the control group as shown in Figure 3.

**Figure 4:**
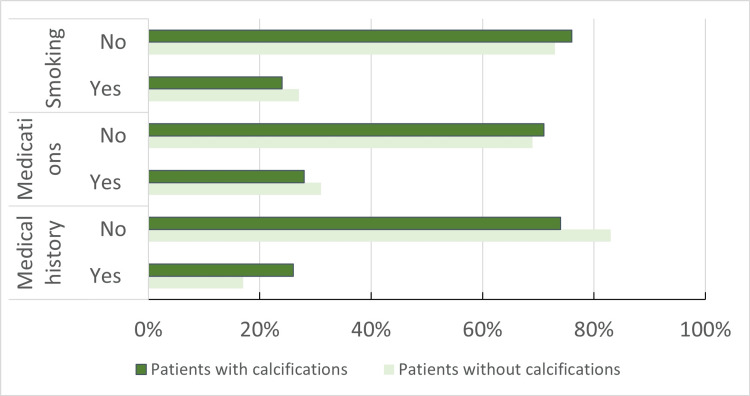
Distribution of smoking, medications, and medical history among patients with calcifications and without calcifications (n=100) in each group

Regarding medication intake, the study did not identify any correlation between certain medications and pulp canal calcification. Among patients without canal calcification, 31 (31%) were found to be taking one or more medications, while 28 (28%) of patients with canal calcification reported the use of medications. However, no statistically significant association was observed between medication intake and the presence of pulp canal calcification in one or more teeth (p=0.125) (Figure 4).

The study findings indicate that smoking status did not demonstrate a significant association with teeth calcifications. Among patients without calcification, 27 (27%) were smokers, while 24 (24%) of patients with calcification were smokers. However, the difference in smoking prevalence between the two groups was not statistically significant (p > 0.05).

## Discussion

Pulp calcifications are asymptomatic and often discovered during routine radiographic examination. Researchers detected pulp calcification with intraoral periapical radiographs, bitewing, and orthopantomography. Pulpal calcifications can only be confirmed if a radiopaque mass is noticed inside the pulp chamber or root canal space on intra or extraoral radiographs when the structures are no less than 200 µm in size [18]. Thus, researchers that apply these radiographic imaging to analyze the prevalence of pulp calcifications in different populations could potentially be inaccurate, and the actual prevalence rate might be greater than the research suggests [19].

Various types of radiographs can be used to determine the presence of calcification. Syrynska et al. investigated the prevalence of pulp calcifications on panoramic radiographs while others used periapical or bitewing radiographs [20-22]. Recently, a study reported the advantages of using cone beam computed tomography (CBCT) for the detection of pulp canal calcification [23]. The present study relied only on conventional intraoral radiographs in terms of detection and evaluating pulp canal calcification. Utilization of CBCT for the sole purpose of evaluating calcification is highly implausible, as these calcifications are unlikely to pose any challenge to the successful completion of the evaluation. Nevertheless, in certain specific cases of complications associated with the treatment, CBCT may prove to be a useful imaging tool during the treatment.

The findings of this study determine the correlation between aging and pulp calcification, our study reported a higher prevalence of 57 (57%) of calcification in patients under 30 years old compared to only 43 (43%) in patients over 30 years old, suggesting no correlation between aging and pulp calcification. This finding is in accordance with a previous study [24]. Accordingly, it can be concluded that the possible causes of pulp calcification are chronic irritation, such as parafunctional habit, deep caries, or restorations other than aging. In addition, frequency, duration, and intensity of the irritant may also be relevant causes. The only effective way to assess the impact of aging on pulpal calcification formation is by enrolling patients in an annual recall visit with radiographic follow-up images. With respect to gender, the prevalence of pulp chamber calcifications in the current study was significantly greater in females 62 (62%) than in males 38 (38%). This finding agrees with the literature [25].

Different studies have shown a correlation between pulp calcification and certain systemic diseases such as cardiovascular disease, diabetes mellitus, and renal lithiasis [10,26]. Further, researchers believe that the detection of pulp calcification can be a diagnostic marker of systemic disease [27]. This study has analyzed certain systemic variables that may correlate to pulp calcification including systemic conditions, medications, and smoking habits. Our study reported that 26 (26%) of patients with medical diseases have one or more teeth with pulp calcification. Furthermore, the most prevalent medical condition in patients with pulp calcification was found in diabetes mellitus by 10 (10%) patients, followed by 4 (4%) in patients with hypertension. Yet, no significant correlation was noted. The reason for the presence of these conditions could be attributed to the latest report involving an age range between 30 to 70 years in the Saudi Arabian population showed the following prevalence of systemic conditions: 26% for hypertension, 23.7% for diabetes, 12.8% for smoking, 10% chronic renal disease [28]. Our study is in accordance with a previous study that evaluated the incidence of pulp calcification in Saudi Arabia's population with diabetes mellitus and cardiovascular diseases using CBCT [29]. The study revealed that pulp calcification was higher in patients with the cardiovascular disease group reaching 32.87%, whereas the diabetes mellitus group exhibited a prevalence of 23.68%. The findings of diabetes mellitus patients with pulp calcifications varied in different studies [29,30].

It is worth mentioning that this study did not show any significant correlation between medications, smoking, and pulp canal calcification. This could be attributed to the type of medications in our study. Previous studies showed a significant correlation between statin and glucocorticoid and canal calcification [11,12]. Yet, our patients were under different medications such as thyroxine, metformin, and glicalazide. which did not show any correlation with pulp calcification.

In regards to smoking, only one study showed a correlation between smokeless tobacco and calcification [17]. Our present study focused only on cigarette smoking. Further information about the type, duration, and frequency of cigarettes should be considered for accurate correlation.

Although the findings of the present study did not reach a statistical significance level, it is important to interpret the findings with caution. As a comparative study between subjects with pulp calcifications and control subjects, there is a potential for overestimation of the risk of pulp calcification development. Furthermore, the present study's case-control design allows for the detection of causal associations between pulp calcification and systemic factors. Nevertheless, no significant association between the systemic factors and canal calcification was found in the current study. As a result of these findings, certain systemic conditions, certain medications, and smoking are not considered to be risk factors for pulp calcification development.

Limitations

The current study has some limitations, including the inclusion of only patients seeking dental treatments which would compromise the types and severity of medical diseases, and not represent the accurate correlation. On the other hand, other systemic diseases such as bone, kidney, joint, and gastrointestinal disorders have not been accounted for. Additionally, in two-dimensional radiography, the structures are superimposed, and the images are distorted, potentially affecting the accuracy of the results. Furthermore, this study design should be designed to conclude the direction of the causal associations between the variables investigated in the current study. Moreover, such a study design will be useful in investigating the relationship between pulp calcification and different inflammatory oral disorders. Furthermore, the effect of medication in various stages of hypertension and diabetes mellitus (well-controlled or poorly controlled) and its association with the incidence of pulp calcifications need to be assessed.

## Conclusions

From the current study it can be concluded that the characteristics of pulp calcification noticed and provided recent insights on its prevalence and the factors related with this condition. A finding of pulp calcification on routine radiographic examination could indicate an underlying unnoticed systemic disorder that demands accurate referral and subsequent therapy. Patients with diabetes mellitus had a higher prevalence of pulp calcification. This study is the baseline for future more intensive studies associating different systemic diseases and medications and pulp canal calcifications.
